# Subacute Sclerosing Panencephalitis after Measles Outbreaks, the Philippines, 2007–2022

**DOI:** 10.3201/eid3211.261395

**Published:** 2026-11

**Authors:** Maela P. Palisoc, Maridel S. Ramones, Roland Dominic G. Jamora

**Affiliations:** National Children’s Hospital, Quezon City, the Philippines (M.P. Palisoc, M.S. Ramones); College of Medicine, San Beda University, Manila, the Philippines (M.P. Palisoc); College of Medicine and Philippine General Hospital, University of the Philippines Manila, Manila (R.D.G. Jamora)

**Keywords:** measles, viruses, subacute sclerosing panencephalitis, SSPE, measles vaccination, vaccination coverage, outbreaks, the Philippines, child neurology

## Abstract

Subacute sclerosing panencephalitis (SSPE) is a delayed, almost uniformly fatal complication of measles. The Philippines had measles outbreaks in 2010–2011, 2013, and 2018–2019; the last was the largest in the World Health Organization Western Pacific Region. Published SSPE data from the Philippines end in 2015. We reviewed all 20 children admitted with SSPE to a tertiary pediatric hospital in Quezon City during 2007–2022 and estimated year of measles infection. Median patient age was 6.2 years at hospital admission and 0.7 years at reported measles illness. Five (25.0%) of 20 admissions occurred in 2022, and estimated infection dates placed 4 patients within the 2018–2019 outbreak; all were admitted during 2020–2022. Given the multiyear latency of SSPE, additional cases might emerge over coming years. Reported measles history could not be established in one quarter of patients. Strengthened measles surveillance and sustained 2-dose vaccination coverage are needed.

Subacute sclerosing panencephalitis (SSPE) is a progressive neurodegenerative disorder caused by persistent central nervous system infection with a defective, hypermutant measles virus. It typically follows measles acquired in early childhood after a latency of years and is almost always fatal (*1*,*2*). Because SSPE follows measles by years rather than weeks, the neurologic consequences of an outbreak do not become visible until long after the outbreak has ended.

Measles vaccination was introduced in the Philippines in 1983; the first routine dose is scheduled at 9 months of age (*3*). Nationwide immunization campaigns in 1998 and 2004 were followed by historically low measles incidence in 2006 (*3*). First-dose measles-containing vaccine coverage later declined from 92% in 2004–2007 to 80% in 2015–2016 and 64% in 2021 (*4*). Reported measles cases rose sharply during a 2018–2019 outbreak, from 2,428 in 2017 to 48,525 in 2019, the largest outbreak in the World Health Organization Western Pacific Region during that period (*5*).

Published data on SSPE in the Philippines are limited; the largest local series described children seen at a single tertiary hospital during 2005–2015 (*6*). No published series from the Philippines spans the period during which children infected in the 2018–2019 outbreak would be expected to demonstrate symptoms of SSPE. We describe children admitted with SSPE to a tertiary government pediatric hospital over 16 years and examine the distribution of their estimated years of measles infection against the national outbreak record.

## Methods

### Design and Setting

We retrospectively reviewed the records of all children admitted to National Children’s Hospital, a tertiary government pediatric hospital in Quezon City, the Philippines, with a final diagnosis of SSPE during January 1, 2007–December 31, 2022. All such patients were enumerated. The 2007 start date reflects the period from which complete institutional records and corresponding registry data were available.

### Case Definition

We modified the case definition from Dyken’s criteria (*1*). We defined a case as a clinical diagnosis of SSPE by the attending pediatric neurologist on the basis of progressive neurologic deterioration with qualitatively positive measles antibody in cerebrospinal fluid (CSF), serum, or both. We excluded children with bacterial, tuberculous, or viral meningitis and children with intracranial masses. Because quantitative measles antibody titers, CSF globulin fractionation, brain biopsy, and uniform electroencephalography were unavailable, we could not apply the full Dyken criteria; we classified cases as clinically diagnosed SSPE with varying levels of supportive evidence.

### Variables

Age at reported measles infection and reported measles history came from parental or guardian recall recorded at admission; measles history was recorded as reported, absent, or unknown. Vaccination status was likewise based on parent or guardian report recorded at admission and was not consistently verified against immunization records.

### Estimation of Year of Reported Measles Infection

For each patient, we calculated the interval between measles infection and development of SSPE as age at hospital admission minus age at measles infection. We obtained the estimated calendar year of measles infection by subtracting that interval from the year of admission. We then compared estimated infection years with the recorded periods of national measles outbreak activity.

For the 14 children with a parent- or guardian-reported history of measles, we calculated the interval between reported measles illness and hospital admission. We excluded 3 children with a reported measles-to-admission interval of <1 year from the interval analysis, because such a short interval is atypical for SSPE and the timing of the reported measles illness could not be independently verified. A very short interval can also reflect acute measles encephalitis (within 14 days of rash onset) or acute disseminated encephalomyelitis (within weeks), rather than SSPE, which follows a latency of years (*1*).

### Statistical Analysis

We summarized categorical variables as frequencies and percentages and summarized continuous variables as medians with interquartile ranges. We did not group patients by admission year for comparison, because the multiyear interval between infection and admission makes such grouping uninterpretable. Analyses used Stata 16.1 (StataCorp LLC, https://www.stata.com) and Microsoft Excel (https://www.microsoft.com). The protocol was approved by the National Children’s Hospital Institutional Review Board (approval no. NCH-IRB 2023–12-NCT) using unlinked, deidentified data.

## Results

Twenty children admitted during January 1, 2007–December 31, 2022, met the case definition. Cases were unevenly distributed across the study period (Figure, panel A). Eight (40.0%) of the 20 admissions occurred in the final 3 years of the series, and 5 (25.0%) admissions occurred in 2022 alone.

**Figure:**
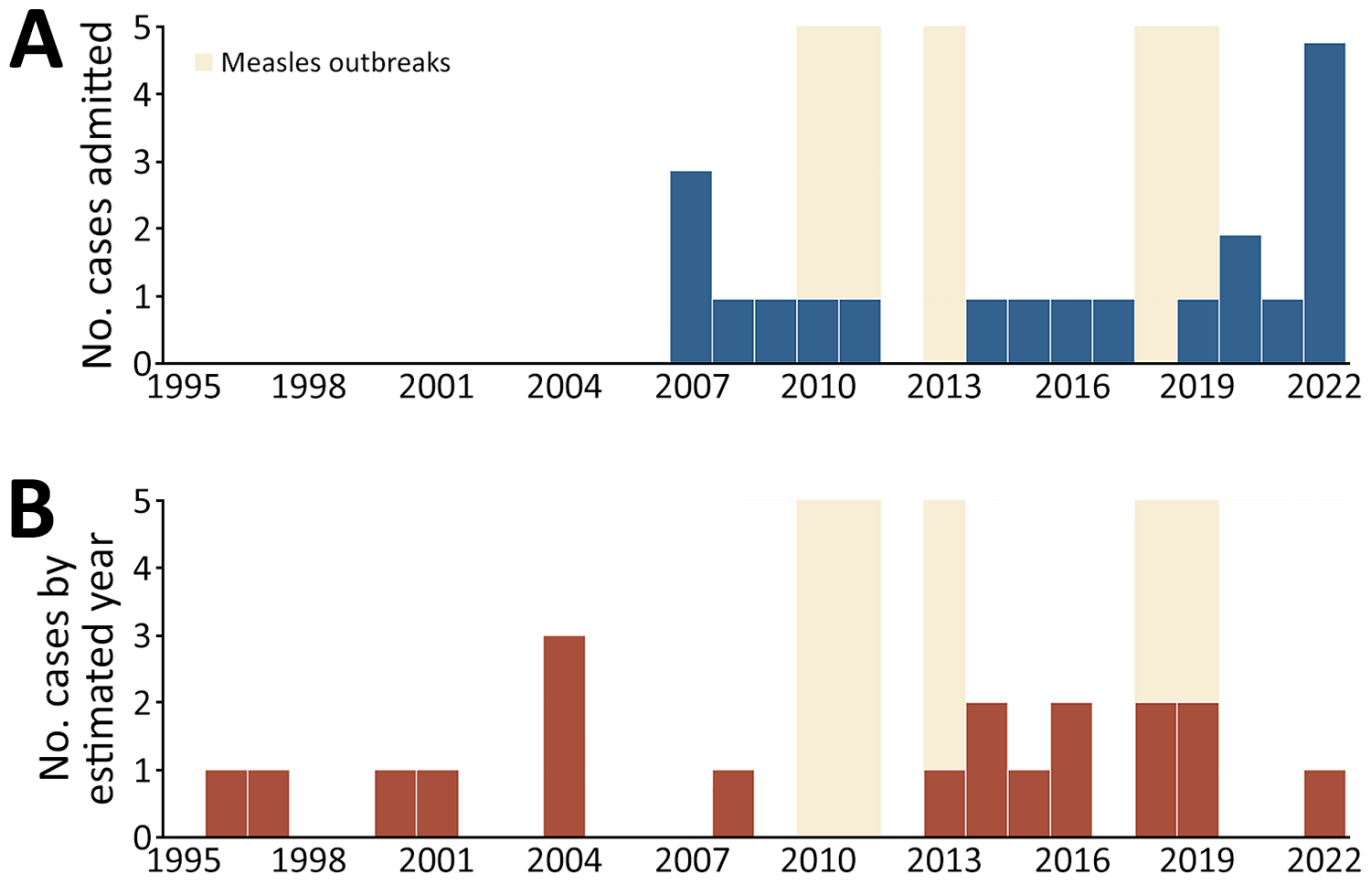
Hospital admissions for subacute sclerosing panencephalitis (SSPE) in study of SSPE after measles outbreaks, Philippines, 2007–2022. A) SSPE admissions by year (blue); B) the same cases by estimated year of measles infection (maroon). Ivory bands mark periods of reported nationwide measles outbreaks (2010–2011, 2013, 2018–2019). For 1 patient, no recorded age at measles infection was available; that patient is not represented in panel B.

Four patients had an estimated measles infection year of 2018 or 2019, corresponding to the national outbreak period (Figure, panel B); all 4 had a reported measles history and positive CSF antibody, and all were admitted during 2020–2022 with intervals of 2.1–3.5 years since measles infection. For 3 patients, the estimated infection year was 2004; estimated infection year for 1 patient was 2013. Most patients with an estimable year (14 of 19) were infected outside recognized outbreak periods, consistent with continued transmission between outbreaks.

SSPE was suspected at time of admission in 9 (45.0%) patients. The remainder were admitted under other working diagnoses: probable intracranial mass in 4 (20.0%) patients, meningitis in 3 (15.0%) patients, encephalitis in 2 (10.0%) patients, and 1 (5.0%) each of acute rheumatic fever and traumatic brain injury. At discharge, 18 (90.0%) patients carried a diagnosis of SSPE and 2 (10.0%) patients probable SSPE.

Of the 20 patients, 14 (70.0%) patients were male and 6 (30.0%) were female. The median age was 6.2 years at admission and 0.7 years at reported measles illness; 10 of the 19 patients with an estimable age were infected before the age of 9 months. Eight children had stunting or wasting, and 3 had comorbidities. A reported measles history was present in 15 (75.0%), unknown in 4 (20.0%), and absent in 1 (5.0%). Seventeen (85.0%) patients had received >1 measles-containing vaccine; 11 (55.0%) patients had received a single dose only. Twelve (60.0%) patients were admitted in stage 2 and 5 (25.0%) were admitted in stage 3 (Table; Appendix Table). Among the 14 children with a reported measles history, the median interval from measles to hospital admission was 5.8 (interquartile range 3.5–8.9) years.

**Table:** Demographic and clinical characteristics of 20 children with subacute sclerosing panencephalitis in study of subacute sclerosing panencephalitis after measles outbreaks, National Children’s Hospital, the Philippines, 2007–2022*

Characteristic	No. patients, N = 20
Sex	
M	14 (70.0)
F	6 (30.0)
Median age at admission, y (IQR)	6.2 (3.8–8.4)
Median age at reported measles illness, y, n = 19 (IQR)	0.7 (0.4–2.0)
Median duration of symptoms before admission, d (IQR)	82 (30–128)
Median length of stay, d (IQR)	10 (6–14)
Nutritional status	
No stunting, no wasting	12 (60.0)
Wasted, not stunted	5 (25.0)
Concurrent stunting and wasting	2 (10.0)
Stunted, not wasted	1 (5.0)
No comorbidity	17 (85.0)
Tuberculosis	2 (10.0)
Bilateral cryptorchidism	1 (5.0)
Reported measles history	
Present	15 (75.0)
Unknown	4 (20.0)
Absent	1 (5.0)
Vaccination	
Measles vaccine only	11 (55.0)
Measles and MMR	6 (30.0)
None	3 (15.0)
Stage at admission†	
Stage 1	3 (15.0)
Stage 2	12 (60.0)
Stage 3	5 (25.0)
Stage 4	0
*Values are no. (%) except as indicated. IQR, interquartile range; MMR, measles, mumps, and rubella. †Clinical stage follows the conventional description of SSPE: stage 1, behavioral and cognitive decline; stage 2, myoclonus and seizures; stage 3, extrapyramidal signs and progressive unresponsiveness; stage 4, coma or vegetative state (*1*,*2*).	

Among the 20 children, 5 had progressive neurologic deterioration, tested positive for measles antibody in CSF, and had characteristic electroencephalography; 12 had progressive deterioration and antibody-positive CSF without electroencephalography; and 3 had progressive deterioration with antibody-positive serum only. Measles antibody testing of CSF was performed in 17 patients and was positive in all 17; serum antibody was tested in 13 and positive in all 13. Both were tested in 10 patients; 3 patients had serum testing alone. Electroencephalography was performed in 5 patients (25.0%); all showed patterns consistent with SSPE (*7*).

## Discussion

In this 16-year series, cases of SSPE clustered in recent years: 40% of admissions occurred in the last 3 years of the study period (2020–2022) and one quarter occurred in 2022 alone; 4 children had an estimated measles infection during the 2018–2019 outbreak. Because SSPE follows measles after a latency of years, those observations most likely represent the leading edge of a postoutbreak wave; additional cases might emerge over the coming years. For public health, the priority is heightened measles surveillance, early recognition of SSPE in children infected during recent outbreaks, and restoration of high 2-dose vaccination coverage.

The short intervals between measles infection and SSPE (2.1–3.5 years) seen in the 4 outbreak-associated cases do not indicate shortening latency: a child infected in 2018 cannot show an interval beyond 4 years in a series closing in 2022, so recent cases are truncated by the observation window. First-dose coverage fell to 64% by 2021, enlarging the susceptible pool beyond the 2018–2019 cohort. A comparable postoutbreak resurgence of SSPE has been reported from the United Kingdom (*8*).

A reported measles history was absent or unknown in a quarter of children; in those children, diagnosis rested on progressive neurodegeneration and presence of measles antibody in CSF or serum. Absence of a reported measles history is often used to argue against SSPE, yet such reasoning would have delayed or missed diagnosis in 5 of those 20 children. Measles antibody testing of CSF is therefore warranted in any child with progressive neurodegeneration and myoclonus, regardless of reported history.

Although 85% of children had received a measles-containing vaccine, that does not implicate vaccination: SSPE follows wild-type measles, not the vaccine (*1*,*2*). Ten of 19 patients were infected before 9 months, the age of the first scheduled measles-containing vaccine dose in the Philippines (*3*) and an age group at particularly high risk for SSPE (*1*,*2*), and most had received only 1 dose. Those findings support earlier and more complete 2-dose coverage and supplementary immunization during outbreaks.

The profile (male predominance, diagnostic delay of weeks to months) was broadly comparable with an earlier Philippines series (*6*); undernutrition and social disadvantage, noted locally (*9*), might limit timely vaccination and thereby raise measles and SSPE risk. SSPE cases in the Philippines currently cannot be staged against international criteria because quantitative antibody titers, CSF globulin fractionation, and consistent electroencephalography and neuroimaging are unavailable, a diagnostic-capacity gap worth closing in a country with recurrent measles outbreaks.

The first limitation of this study is that, as a single-center retrospective review of 20 patients, it might not represent SSPE nationally, and no population denominator was available, so counts cannot be expressed as rates. Second, measles history, age at infection, and vaccination status were based on guardian recall and could not be verified; the infection-to-SSPE interval is therefore estimated and could not be determined in one quarter of children. Measles antibody was reported only qualitatively, and 3 children were diagnosed using serum antibody without CSF confirmation, although the temporal findings were unchanged when restricted to the 17 with CSF confirmation. Finally, molecular confirmation and genotyping, which require tissue, were not performed, and outcome after discharge was not available.

In conclusion, measles antibody testing of CSF should be considered in any child with progressive neurodegeneration and myoclonus, irrespective of the reported measles history. Additional SSPE cases temporally associated with the 2018–2019 outbreak might continue to occur over the remainder of the 2020s. National surveillance for SSPE should be strengthened accordingly. Laboratory capacity should be developed for quantitative measles antibody titration in serum and CSF and for CSF globulin fractionation, so that Philippine cases can be characterized against international diagnostic criteria. Prospective studies capturing outcome and survival in children from the Philippines with SSPE are needed, as is evaluation of supportive and disease-modifying treatment in this population, building on the limited local experience with intravenous immunoglobulin (*10*). Efforts to restore and sustain 2-dose measles vaccination coverage, including supplementary immunization activities during outbreaks, remain the only means of preventing disease. Where postmortem or biopsy tissue is available, molecular confirmation of measles RNA and genotyping to link cases to circulating strains would strengthen future SSPE surveillance. 

Appendix Additional information about subacute sclerosing panencephalitis after measles outbreaks, the Philippines, 2007–2022.
