# Supplementary material for: Subacute Sclerosing Panencephalitis after Measles Outbreaks, the Philippines, 2007–2022
Source: Emerg Infect Dis. 2026 Nov;32(11):1793–7. doi: 10.3201/eid3211.261395 (PMC13715892; doi:10.3201/eid3211.261395)
Supplement: Supplementary file 1 [file 26-1395-Techapp-s1.pdf]

Article DOI: https://doi.org/10.3201/eid3211.261395

EID cannot ensure accessibility for supplementary materials supplied by authors. Readers who have difficulty accessing supplementary content should contact the authors for assistance.

Subacute Sclerosing Panencephalitis after Measles Outbreaks, the Philippines, 2007–2022

Appendix

Appendix Table. Individual characteristics of 20 children with subacute sclerosing panencephalitis, National Children's Hospital, Philippines, 2007–2022.

Sex	Age at reported measles (y)	Reported measles / est. year	Vaccination	Age at admission (y)	Interval (y)	Stage	CSF Ab	Serum Ab	EEG	Criteria met
F	0.7	reported / 1996	measles only	15.3	14.6	3	+	+	ND	2
M	2.0	reported / 1997	measles+MMR	12.1	10.1	2	+	+	ND	2
M	2.0	reported / 2000	none	11.6	9.6	2	+	ND	ND	2
M	0.5	reported / 2001	none	6.8	6.3	2	+	+	ND	2
M	0.8	reported / 2004	none	4.3	3.5	2	+	+	ND	2
M	1.0	reported / 2004	measles only	6.3	5.3	2	+	ND	ND	2
F	0.7	reported / 2004	measles only	11.4	10.7	3	+	ND	typical	3
M	0.6	reported / 2013	measles only	6.2	5.6	3	+	+	typical	3
F	0.4	reported / 2014	measles only	6.3	5.9	2	+	+	typical	3
F	0.4	reported / 2014	measles only	7.4	7.0	2	ND	+	ND	1
F	3.3	absent / 2016	measles only	3.5	0.2	3	ND	+	ND	1
M	0.2	reported / 2018	measles only	2.3	2.1	2	+	ND	typical	3
M	0.3	reported / 2018	measles+MMR	3.8	3.5	1	+	ND	ND	2
M	0.3	reported / 2019	measles+MMR	3.2	2.9	1	+	+	ND	2
M	0.4	reported / 2019	measles+MMR	3.6	3.2	2	+	ND	ND	2
M	5.3	unknown	measles only	5.8	0.5	3	+	+	typical	3
M	NR	reported / NR	measles only	11.6	NR	1	+	ND	ND	2
M	2.0	unknown	measles only	4.1	2.1	2	+	+	ND	2
F	1.0	unknown	measles+MMR	6.8	5.8	2	ND	+	ND	1
M	3.3	unknown	measles+MMR	3.4	0.1	2	+	+	ND	2

Reported measles / est. year: parent- or guardian-reported measles history and the estimated calendar year of infection ("unknown" where the history could not be established). Interval: years from reported measles illness to hospital admission. Criteria met: number of assessable supportive features present — progressive neurologic deterioration (present in all by inclusion), cerebrospinal fluid measles antibody, and characteristic electroencephalography. +, positive; ND, not done; NR, not recorded. Age at vaccination was not recorded in the source data.
